# Adenoma Characteristics and the Influence of Alcohol and Cigarette Consumption on the Development of Advanced Colorectal Adenomas

**DOI:** 10.3390/ijerph17228296

**Published:** 2020-11-10

**Authors:** Maja Čebohin, Senka Samardžić, Ksenija Marjanović, Martina Tot Vesić, Kristina Kralik, Andreja Bartulić, Dijana Hnatešen

**Affiliations:** 1 Medicinska škola Osijek, 31000 Osijek, Croatia; martina.tot@skole.hr; 2 Faculty of Medicine, Josip Juraj Strossmayer University of Osijek, 31000 Osijek, Croatia; senka.002@gmail.com (S.S.); marjanovic.ksenija@kbco.hr (K.M.); kristina.kralik@gmail.com (K.K.); hnatesen.dijana@kbco.hr (D.H.); 3 Department of Nursing and Palliative Medicine, Faculty of Dental Medicine and Health Osijek, Josip Juraj Strossmayer University of Osijek, 31000 Osijek, Croatia; 4 Institute of Public Health Osijek–Baranja County, 31000 Osijek, Croatia; 5 Clinical Department of Pathology and Forensic Medicine, University Hospital Osijek, 31000 Osijek, Croatia; 6 Clinical Department of Gastroenterology and Hepatology, University Hospital Osijek, 31000 Osijek, Croatia; andrejaor@gmail.com; 7 Clinical Department of Pain Management, University Hospital Osijek, 31000 Osijek, Croatia

**Keywords:** cancer prevention, advanced adenoma, colorectal adenoma, alcohol consumption, cigarette smoking

## Abstract

*Background*: Colorectal cancer (CRC), one of the leading public health problems worldwide, is a disease that can be prevented when it is detected in time. The objectives of this cross-sectional study were to investigate the characteristics of colorectal adenomas and whether alcohol consumption and cigarette smoking correlated with the development of advanced adenomas in participants in The National Programme for Early Detection of Colorectal Cancer (NP) in Osijek-Baranja County (OBC), Croatia. *Methods*: The screening methods were the guaiac Faecal Occult Blood Test (gFOBT), colonoscopy, histological analysis, and risk factor questionnaire. *Results*: The results showed the presence of adenomas in 136 men (57.4%) and 101 women (42.6%), *p* < 0.001. There was one adenoma in 147 (62%) most commonly located in sigmorect, in 86 (59%) participants, and 44 (18.6%) participants had multiple adenomas, most commonly found in multi loc, *p* < 0.001. According to size, 118 (49.8%) of all adenomas were between 0.1 and 0.9 cm, while adenomas of 3 cm 19 (8%) were the fewest, *p* < 0.001. There were 142 (59.9%) advanced adenomas. *Conclusions*: Adenoma development in the OBC population was correlated with predictors: adenoma size, high-grade dysplasia, smoking and alcohol consumption of 20 g per day. Non-smoking was found to be a health protective behaviour.

## 1. Introduction

CRC one of the leading public health problems worldwide, is a disease that can be prevented when it is detected in time. According to Global Cancer Incidence, Mortality and Prevalence (GLOBOCAN), CRC was the third most common cancer in men, after lung cancer and prostate cancer, while in women it was the second most common cancer, after breast cancer, but a number one cause of death [1]. A high incidence rate was recorded in Australia, New Zealand, North America and Europe, while the rate was lower in Africa and South and Central Asia [2]. According to the Croatian Cancer Registry, there were 2143 (16%) CRCs in men and 1516 (14%) in women in 2017 [3]. It is widely accepted that most CRCs arise from adenomas (adenoma–carcinoma sequence) [4]. Specific adenoma characteristics are related with higher risks for CRC development: size (≥10 mm), villous histology, and high-grade dysplasia.

The development of advanced adenomas and carcinomas is associated with behavioural risk factors that were researched in many studies, and the following were identified: cigarette smoking [5,6], obesity [7,8,9], red meat and processed meat consumption [10,11], and alcohol consumption [12,13]. The World Cancer Fund and American Institute for Cancer Research data analysis showed that consuming more than 30 g/day of ethanol in alcoholic drinks is a convincing risk factor for the development of CRC in men and a probable risk factor in women [14]. The International Agency for Research on Cancer (IARC) included colorectal cancer in a list of cancers related to alcohol consumption [15]. Cigarette smoking is confirmed as a risk factor for the development of adenomas and advanced adenomas in both men and women [16]. In the 2012 IARC Volume 100 E, cigarette smoking was recognized as a cause of colorectal cancer [17].

A meta-analysis of the data obtained in 106 observation studies showed a greater risk for CRC development in smokers than in nonsmokers [18]. As there is greater risk for the development of colorectal adenomas in smokers, some authors suggest that smokers should have screening examinations earlier than nonsmokers [19,20]. The CRC incidence varies significantly, depending on geographic position, genetics, and lifestyle [21,22]. This study was conducted in OBC situated in the northeast of Croatia, covering an area of 4152 km^2^, with a predominantly rural population whose lifestyle is specific and diet is characterized by the overconsumption of fat and refined carbohydrates.

According to data obtained in the 2003 Croatian Adult Health Survey (CAHS), the highest prevalence of poor dietary habits was found in eastern and central Croatia (23.8% and 23.0%, respectively) [23]. OBC suffered devastation during the Croatian Homeland War in the 1990s, and this had a great impact on people’s lifestyle and habits (including alcohol consumption and smoking). The results of the CAHS showed that the highest prevalence of alcohol consumption (14.09%) was recorded in the male population in the eastern region [24], as well as the highest prevalence of smoking (33.10%) [25]. The purpose of this study was to investigate characteristics of colorectal adenomas, and whether alcohol consumption and cigarette smoking correlated with the development of advanced adenomas in participants in the NP in OBC. Identification of probable risk factors related to the development of colorectal adenomas helps in the prevention of colorectal cancers. Primary prevention is focused on the recognition and removal of environmental carcinogens and their metabolic activation, as well as the monitoring of genetic factors, while secondary prevention includes all interventions that can be used to diagnose the disease at early, curable stages or to diagnose premalignant lesions [26]. Treatment options for advanced CRC are limited; therefore, more attention should be directed toward early detection and prevention [27].

## 2. Materials and Methods

The target population in this study were the participants in the NP in OBC, from December 2013 to July 2017, in the 50–74-years-of-age group, considered at average risk, whose haemoccult test was positive. The test used in this study was the gFOBT. This testing requires three gFOBT cards, consisting of four separate slots to smear a stool sample on a test card. The samples are taken from three consecutive stools. If one of the samples is positive for blood, the participant is referred for a colonoscopy. The participants received a letter explaining the procedure and a written consent form and were asked to fill it in and return it to the Institute of Public Health of OBC by mail. Then, having signed the consent, the participants received three test cards and instructions on how to use them. Test cards with stool samples were then sent back to the Institute by mail and analysed.

The participants whose test results were positive for blood were referred to the University Hospital Osijek for colonoscopy. During the colonoscopy, all polypoid lesions were removed and then analysed at the Clinical Institute of Pathology and Forensic Medicine, University Hospital Osijek. The histological classification of polyps and cancers is based on World Health Organization (WHO) criteria [28]. Out of the adenomatous polyps, advanced adenomas were defined by the presence of the following components: size (≥10 mm), histological grade (high-grade dysplasia), and the degree of villous component (>20%) [29,30]. The malignancy risk in adenomatous polyps correlates with the size, histological type, and dysplasia grade [31].

In participants with adenomas and advanced adenomas, further analysis of their answers in a NP structured questionnaire was performed regarding the risk factors for CRC development. Special attention was given to items regarding alcohol and cigarette consumption: whether a participant consumes alcohol (yes/no), the amount of alcohol consumed per day (≤10 g, ≤20 g, and 30 g or more), whether a participant was a smoker (yes/no/former smoker), the number of cigarettes smoked per day (≤10, ≤20, and 21 or more), the years of smoking (≤10, ≤20, and 21 or more). The categorical data are presented using absolute and relative frequencies. The categorical data differences were tested using the χ^2^ test, and Fisher’s Exact Test, as necessary (when in m x n table the expected frequency is less than 5 in more than 20% of cases). The post hoc analyses were carried out taking into account Bonferroni or Sidak correction to correct the level of significance, considering that we were carrying out multiple comparisons. Multivariate logistic regression (stepwise method) was applied to derive a prediction model for advanced adenomas. All p values are two-sided. The level of significance, alpha (α), was set at 0.05. MedCalc Statistical Software version 18.11.3 (MedCalc Software bvba, Ostend, Belgium) and SPSS Statistics 23 (IBM Corp., Released 2015. IBM SPSS Statistics for Windows, Armonk, NY, USA) were used for statistical analysis.

Ministry of Health of the Republic of Croatia approved this study (Approval number: 053-02/19–01/623) and ethical committee of Osijek University Hospital (Approval number: R1-1186-2/2019).

## 3. Results

Within the NP in the Republic of Croatia, 688 persons, aged 50–74 years, in OBC were invited to participate in the period from December 2013 to July 2017. The screening tool was gFOBT, and 86.19% of invitees returned their test kit, and 94.3% of those underwent colonoscopy. An adenomatous polyp was identified in 42.4% of participants.

### 3.1. Distribution of Participants with Adenomas According to Sex and Age

The number of colorectal adenomas in men aged 50–59, 73 (53.7%), was significantly greater than the number in the 60–69 age range, while in women more adenomas were found in the 60–69 age group (50.5%) than in women aged 50–59. There was a significant difference in distribution of participants according to gender and age (Chi-square test, *p* < 0.001) (Table 1).

### 3.2. Distribution of Participants According to the Number of Adenomas and Their Location

There was one adenoma in 147 (62%) participants, and 44 (18.6%) participants had multiple adenomas. One adenoma was most commonly located in sigmorect, in 86 (59%) participants, while two or more adenomas were most commonly found in multi loc. There was a significant difference in distribution of participants according to the number of adenomas and their location (Fisher’s Exact Test, *p* < 0.001) (Table 2).

### 3.3. Distribution of Participants According to Size and Location of Adenomas

According to size, 118 (49.8%) of all adenomas were between 0.1 and 0.9 cm, while adenomas of 3 cm 19 (8%) were the fewest. According to location, 48 (41%) adenomas between 0.1 and 0.9 cm and 12 (63.2%) adenomas of 3 cm were found in sigmorect. There was a significant difference in distribution of participants according to location of adenomas and size (Fisher’s Exact Test, *p* < 0.001) (Table 3).

### 3.4. Distribution of Participants According to Grade and Location

According to grade, most adenomas are classified as low-grade dysplasia 188 (79.3%), with no significant difference regarding localization. (Table 4).

### 3.5. Advanced Adenomas

Tubular adenomas were the most common type (82.3%), while tubulovillous adenomas were found in 15.2% of participants, villous in 0.8% of participants, and a combination of tubular and tubulovillous adenomas in 1.7% of participants. Advanced adenomas were found in 59.9% of participants, mostly due to their size (50.2%). High-grade dysplasia was found in 20.7% of adenomas, while a villous component greater than 20% was present in 18.6% of adenomas (Table 5).

### 3.6. Comparison of Habits in Participants with Adenomas and Advanced Adenomas—Alcohol and Cigarette Consumption

There was significant difference in distribution of participants with adenomas and advanced adenomas according to the amount of alcohol they consume (Fisher’s Exact Test, *p* = 0.03), cigarette smoking (Chi-square test, *p* = 0.02) and number of cigarettes per day (Chi-squared test, *p* = 0.01). A significantly greater number of participants with adenomas consume one or two glasses, up to 10 g of alcohol, in comparison to participants with advanced adenomas (34% or 81% and 41% or 57.7%, respectively). Fewer participants with advanced adenomas were smokers, compared to participants with adenomas (24% or 16.9% and 30% or 31.6%, respectively). According to the number of cigarettes per day, a significantly greater number of participants with advanced adenomas (10% or 18.5%) smoke 21 or more cigarettes, in comparison to participants with adenomas (Table 6).

### 3.7. Predicting the Probability of Developing Advanced Adenomas

We used logistic regression to evaluate the influence of multiple factors on the probability of developing advanced adenomas (dependent variable). Sex, age, location, size, grade, alcohol and cigarette consumption were used as independent variables. Multivariate logistic regression was applied to derive a prediction model for advanced adenomas.

The results showed that four independent predictors had statistically significant contributions to the prediction model (size, high-grade dysplasia, alcohol consumption, and non-smoking). The entire model was significant (χ^2^ = 60.3, *p* < 0.001). Variance in the present advanced adenomas was explained by these predictors in 41.4% of the cases according to Cox and Snell, and in 56.5% of the cases according to Negelkerke, while classification was accurate in 83.2% of the cases. The strongest predictors were adenoma size (odds ratio (OR) is 25.6) and grade (OR is 19.6), while nonsmoking was a health protective behaviour (OR is 0.13) (Table 7).

## 4. Discussion

The population of OBC that participated in the NP had the second highest incidence of colorectal cancer in the Republic of Croatia, after the City of Zagreb region, in the period from 2007 to 2011 [32]. The research carried out in OBC during the first six years of the NP implementation, until February 2013, showed a large number of diagnosed colorectal cancers and advanced adenomas [33]. Significantly more colorectal adenomas diagnosed in this study were present in men, 57.4%, which complies with other studies [34,35]. The incidence of adenomas among male participants in this study was highest in the age group 50–59 (53.7%), which is corroborated by the results of a study carried out in Teheran [34]. In women, the highest incidence of adenomas was in the age group 60–69 (50.5%). Most adenomas diagnosed in this study were 0.1–0.9 cm (49.8%), which complies with the results obtained in an American study conducted by specialized gastroenterological laboratory Miraca Life Sciences [36].

Advanced adenomas are the research topic of numerous studies, as their potential to develop into colorectal cancer has been recognised. Large adenomas, >3 cm, were detected in 8% of cases in this study, more commonly with high-grade dysplasia (63.2%), in the rectosigmoid colon. The correlation between size and high-grade dysplasia in adenomas was confirmed in research by O’Brien at al. [37]. Advanced adenomas with 1, 2, or 3 components were found in 59.9% of participants in this study, in most cases because of their size (≥1 cm, 50.2%), high-grade dysplasia (20.7%), and villous component >20% (18.6%). Adenoma sizes of 1–1.9 cm and high-grade dysplasia were identified by regression analysis as predictors of developing advanced adenomas. In our study, adenoma size and high-grade dysplasia were important prognostic markers for advanced adenomas development, which is in agreement with other studies [38,39,40,41].

There are reports regarding the increased number of diagnosed CRCs in eastern countries, which is linked to industrialization and changes in living habits [42,43,44,45,46]. The correlation between smoking and the development of pre-cancerous colorectal lesions in both men and women was confirmed in numerous studies [47,48,49], which is in agreement with our study. The participants of this study with diagnosed advanced adenomas smoked more cigarettes per day than participants with adenomas. In this study, regression analysis identified nonsmoking as a health protective behaviour. The participants with advanced adenomas consume larger quantities of alcohol than participants with adenomas. In this study, regression analysis showed that alcohol consumption of more than 20 g per day was a predictor of developing advanced adenomas. These results agree with other studies indicating a correlation between alcohol consumption and colorectal carcinomas in men and women [47,50,51].

This study has certain limitations as it was conducted in one county in the Republic of Croatia and only two behavioural risk factors (alcohol consumption and cigarette smoking) were investigated. Therefore, subsequent studies should include populations from various regions in Croatia, as well as other already identified risk factors of developing advanced adenomas and colorectal carcinomas. Furthermore, the results showed large OR with correspondingly large CI, which is the consequence of a small sample size in this study, and such large effects would be avoided with larger sample size. Increasing the sample size would increase the precision in 95% CI of OR.

## 5. Conclusions

This study corroborated findings that larger adenomas and high-grade dysplasia adenomas correlated with the development of advanced adenomas. Adenoma size of 1–1.9 cm, high-grade dysplasia, consuming 20 g of alcohol per day, and smoking were identified by multivariate analysis as predictors for developing advanced adenomas in OBC, while nonsmoking was a health protective behaviour.

## Figures and Tables

**Table 1:** The distribution of the participants with adenomas according to sex and age.

Age	Number (%) of Participants according to Sex			*p* *
	Men	Women	Total	
50–59	73 (53.7)	23 (22.8)	96 (40.5)	<0.001
60–69	14 (10.3)	51 (50.5)	65 (27.4)	
70–74	49 (36)	27 (26.7)	76 (32.1)	
Total	136 (100)	101 (100)	237 (100)	

* Chi-square test.

**Table 2:** The distribution of participants according to the number of adenomas and their location.

Location	Number (%) of Participants according to the Number of Adenomas				*p* *
	One Adenoma	Two Adenomas	Multiple	Total	
Ascending	20 (13.6)	1 (2.2)	1 (2.3)	22 (9.3)	<0.001
Transverse	16 (10.9)	4 (8.7)	0	20 (8.4)	
Descending	22 (15)	1 (2.2)	1 (2.3)	24 (10.1)	
Sigmorect	86 (59)	14 (30)	3 (7)	103 (43.5)	
Multi Loc	3 (2)	26 (57)	39 (89)	68 (28.7)	
Total	147 (100)	46 (100)	44 (100)	237 (100)	

* Fisher’s Exact Test.

**Table 3:** The distribution of participants according to the size and location of adenomas.

Location	Number (%) of Participants according to the Size of Adenomas					*p* *
	0.1–0.9 cm	1–1.9 cm	2–2.9 cm	3 cm	Total	
Ascending	15 (12.7)	6 (7.9)	0	2 (10.5)	23 (9.7)	<0.001
Transverse	12 (10.2)	4 (5.3)	3 (12.5)	1 (5.3)	20 (8.4)	
Descending	16 (13.6)	5 (6.6)	1 (4.2)	2 (10.5)	24 (10.1)	
Sigmorect	48 (41)	32 (42)	11 (46)	12 (63.2)	103 (43.5)	
Multi Loc	27 (23)	29 (38)	9 (38)	2 (10.5)	67 (28.3)	
Total	118 (100)	76 (100)	24 (100)	19 (100)	237 (100)	

* Fisher’s Exact Test.

**Table 4:** The distribution of participants according to the grade and location.

Location	Number (%) of Participants according to Grade			*p* *
	Low Grade	High Grade	Total	
Ascending	21 (11.2)	2 (4.1)	23 (9.7)	0.23
Transverse	18 (9.6)	2 (4.1)	20 (8.4)	
Descending	18 (9.6)	6 (12.2)	24 (10.1)	
Sigmorect	76 (40)	27 (55)	103 (43)	
Multi Loc	55 (29)	12 (24)	67 (28)	
Total	188 (100)	49 (100)	237 (100)	

* Fisher’s Exact Test.

**Table 5:** Advanced adenomas.

Advanced Adenomas	Number (%)
Size ≥ 1 cm	119 (50.2)
High-grade dysplasia	49 (20.7)
Villous component greater than 20%	44 (18.6)
Advanced adenomas with 1, 2, or all 3 components	142 (59.9)

**Table 6:** Comparison of habits in participants with adenomas and advanced adenomas—alcohol and cigarette consumption.

Alcohol-Cigarette Consumption	Number (%)			*p* *
	Adenomas	Advanced Adenomas	Total	
Alcohol				
Yes	42 (44.2)	71 (50)	113 (47.7)	0.38
No	53 (55.8)	71 (50)	124 (52.3)	
Total	95 (100)	142 (100)	237 (100)	
Amount of alcohol consumed				
1–2 glasses, up to 10 g	34 (81)	41 (57.7)	75 (66.4)	0.03
2–4 glasses, up to 20 g	8 (19)	26 (36.6)	34 (30.1)	
>4 glasses, >30 g	0	4 (5.6)	4 (3.5)	
Total	42 (100)	71 (100)	113 (100)	
Smoking				
No	53 (55.8)	88 (62)	141 (59.5)	0.02
Yes	30 (31.6)	24 (16,9)	54 (22.8)	
Former smokers	12 (12.6)	30 (21.1)	42 (17.7)	
Total	95 (100)	142 (100)	237 (100)	
Cigarettes per day				
Up to 10	12 (28.6)	16 (29.6)	28 (29.2)	0.01
Up to 20	30 (71.4)	28 (51.9)	58 (60.4)	
21 or more	0	10 (18.5)	10 (10.4)	
Total	42 (100)	54 (100)	96 (100)	
Years of smoking				
Up to 10	0	1 (1.9)	1 (1)	0.15 ^†^
Up to 20	16 (38.1)	29 (53.7)	45 (46.9)	
21 or more	26 (61.9)	24 (44.4)	50 (52.1)	
Total	42 (100)	54 (100)	96 (100)	

* χ^2^ test; ^†^ Fisher’s Exact Test.

**Table 7:** Predicting the probability of developing advanced adenomas (multivariate logistic regression—stepwise method).

Predictor	β	Standard Error	Wald	*p*-Value	Odds Ratio (OR)	95% Confidence Interval (CI)
Size (1–1.9 cm)	3.24	0.83	15.1	<0.001	25.6	4.9 to 131.4
Grade (high)	2.97	1.16	6.56	0.01	19.6	2.01 to 191.2
Alcohol consumption (20 g alcohol/day)	2.0	0.67	9.05	0.003	7.4	2.01 to 27.3
Smoking (nonsmoker vs. smoker)	−2.02	0.67	9.14	0.003	0.13	0.04 to 0.49
Constant	−0.50	0.35	2.06	0.15		

β-represents regression coefficient Wald or Wald test represents the significance of the regression coefficient.
